# Effect of the General Anaesthetic Ketamine on Electrical and Ca^2+^ Signal Propagation in *Arabidopsis thaliana*

**DOI:** 10.3390/plants13060894

**Published:** 2024-03-20

**Authors:** Andrej Pavlovič, Lucie Ševčíková, Martin Hřivňacký, Marek Rác

**Affiliations:** Department of Biophysics, Faculty of Science, Palacký University, Šlechtitelů 27, CZ-783 71 Olomouc, Czech Republic; lucie.sevcikova01@upol.cz (L.Š.); martin.hrivnacky@upol.cz (M.H.); marek.rac@upol.cz (M.R.)

**Keywords:** *Arabidopsis*, anaesthetic, calcium, diethyl ether, jasmonates, ketamine, systemic response

## Abstract

The systemic electrical signal propagation in plants (i.e., from leaf to leaf) is dependent on GLUTAMATE RECEPTOR-LIKE proteins (GLRs). The GLR receptors are the homologous proteins to the animal ionotropic glutamate receptors (iGluRs) which are ligand-gated non-selective cation channels that mediate neurotransmission in the animal’s nervous system. In this study, we investigated the effect of the general anaesthetic ketamine, a well-known non-competitive channel blocker of human iGluRs, on systemic electrical signal propagation in *Arabidopsis thaliana*. We monitored the electrical signal propagation, intracellular calcium level [Ca^2+^]_cyt_ and expression of jasmonate (JA)-responsive genes in response to heat wounding. Although ketamine affected the shape and the parameters of the electrical signals (amplitude and half-time, t_1/2_) mainly in systemic leaves, it was not able to block a systemic response. Increased [Ca^2+^]_cyt_ and the expression of jasmonate-responsive genes were detected in local as well as in systemic leaves in response to heat wounding in ketamine-treated plants. This is in contrast with the effect of the volatile general anaesthetic diethyl ether which completely blocked the systemic response. This low potency of ketamine in plants is probably caused by the fact that the critical amino acid residues needed for ketamine binding in human iGluRs are not conserved in plants’ GLRs.

## 1. Introduction

Plants are sessile organisms constantly exposed to environmental perturbation. Mechanical injury or wounding is one of the severest environmental stresses to which plants are subjected in nature. Plants have evolved mechanisms to withstand such stress by activating a defence response mediated by the plant hormones, jasmonates [1,2]. The response is not limited to the organs that initially sense the stress but quickly spreads from damaged tissue to distal parts of the plant that have not yet directly experienced the stimuli during the process termed systemic response. The meaning of systemic signalling is to prepare a multicellular organism for an upcoming challenge that is initially sensed by a single organ or only a small group of cells. These signals include hormones, peptides, volatile compounds, reactive oxygen species (ROS), electrical and Ca^2+^ signals and they often co-propagate together [3,4,5,6,7,8]. Systemic electrical signals have attracted the attention of scientists since their initial discovery [9] and rapid progress has been made in understanding their molecular mechanisms during recent years [4,7]. 

It was quite a big surprise when glutamate receptor-like proteins (GLRs) were discovered in plants [10]. The GLRs are the homologous proteins to animal ionotropic glutamate receptors (iGluRs) which are ligand-gated non-selective cation channels that mediate neurotransmission in the animal nervous system. They share a similar architecture with the extracellular amino-terminal domain (ATD), a ligand-binding domain (LBD), three transmembrane helices (M1, M3, M4) plus a partial one (M2), and a cytoplasmic terminal domain (CTD) [10,11,12]. The degree of identity between *Arabidopsis thaliana* GLRs and animal iGluRs within these domains is 63% to 16% and is similar to that between animal iGluR subtypes that have kainate/AMPA rather than NMDA as agonists, suggesting a divergence of the iGluRs/GLRs prior to their subtype differentiation [10]. Recent studies showed that GLRs play an indispensable role in systemic electrical and Ca^2+^ signal propagation in the plant *A. thaliana*. In particular, the double mutants *glr3.3* and *glr3.6* were not able to propagate electrical and Ca^2+^ signals from local damage to systemic leaves [4,7]. The GLR3.3 and GLR3.6 are expressed and localised in vascular tissue (sieve elements and xylem contact cells, respectively) supporting the role of vasculature in electrical signal propagation in plants [13]. In the glutamatergic synapse of the animal neuron, glutamate is released from the presynaptic neuron, binds to iGluRs on the postsynaptic neurons and activates them, allowing the passage of Na^+^ and Ca^2+^ ions. A simple analogy exists in plants; wounding releases glutamate in the apoplast that binds and activates GLRs [7,14,15,16,17,18,19]. Then, the Ca^2+^ wave is likely driven by the bulk flow of a glutamate or glutamate is released in the systemic leaves by an unidentified mechanism [19,20]. This analogy is further supported by the fact that the exogenous application of glutamate, but not other amino acids, triggers systemic cytosolic calcium ([Ca^2+^]_cyt_) increase [7,15]. However, the crystal structure of the ligand-binding domain (LBD) of AtGLR3.3 reveals the accommodation of a wider range of amino acids [14].

Recently, we showed that the volatile general anaesthetic (GVA) diethyl ether completely inhibited electrical and Ca^2+^ signal propagation from damaged to neighbouring systemic leaves in response to heat wounding in *A. thaliana*. The same inhibition was also found in response to exogenous glutamate application, indicating that GLR channels are among the possible targets of diethyl ether anaesthesia. As a consequence, the anaesthetised plant was not able to activate defence response mediated by jasmonates in systemic leaves [21]. The inhibitory effect of the GVA diethyl ether on electrical (action potentials), Ca^2+^ signal propagation, and downstream responses was also documented in the carnivorous plant, Venus flytrap (*Dionaea muscipula*). This plant captures insects using modified leaves in the traps. Sensitive trichomes (trigger hairs) on the upper trap epidermis function as mechanosensors and when the trigger hairs are stimulated by touch, an action potential (AP) is generated which propagates across the trap lobes. Two touches and the generation of two APs are necessary for rapid trap closure. However, under diethyl ether anaesthesia, the propagation of APs across the lobes is completely blocked [22,23]. Using the transgenic *D. muscipula* expressing calcium sensor GCaMP6f, it was documented that the calcium signal was generated in the trigger hair but could not propagate to the trap lobe under anaesthesia [24]. This resembles the effect of GVA on mammalian neurons, i.e., complete inhibition of AP propagation [25]. Thus, plants under anaesthesia are not able to sense and/or react to certain external stimuli.

Despite the fact that GVAs are used on a daily basis in thousands of hospitals, scientists and the doctors who administer these compounds still lack a molecular understanding of their action. It is still not entirely clear if lipophilic GVAs work nonspecifically by dissolving in the lipid bi-layer (lipid theory of general anaesthesia, [26,27,28]), indirectly affecting ion-channel activity, and/or by binding to the non-specific hydrophobic cavities of many proteins (protein theory of anaesthesia; [29,30,31,32]). On the other hand, the molecular targets of many local and intravenous general anaesthetics are more specific and well-known including GABA, NMDA, sodium and two-pore potassium channels [33]. The intravenous general anaesthetic ketamine is a highly lipophilic compound that easily crosses the membranes and results in the rapid onset of anaesthesia. It is well-known as a non-competitive channel blocker of animal glutamate-gated calcium-permeable NMDA receptors [34]. Due to the sequence homology, the identical structural and functional domains with plant GLRs [10,12], and the indispensability of GLRs in systemic response [4,7], we decided to investigate the effect of ketamine on electrical and Ca^2+^ signal propagation in *A. thaliana* in response to heat wounding. Our study showed that ketamine, in contrast to GVA, was not able to block electrical and Ca^2+^ signal propagation and only slightly modified the characteristics of electrical signals.

## 2. Results

### 2.1. Anaesthetic Ketamine Modified Electrical Signals Mainly in Systemic Leaves

**Figure 1:**
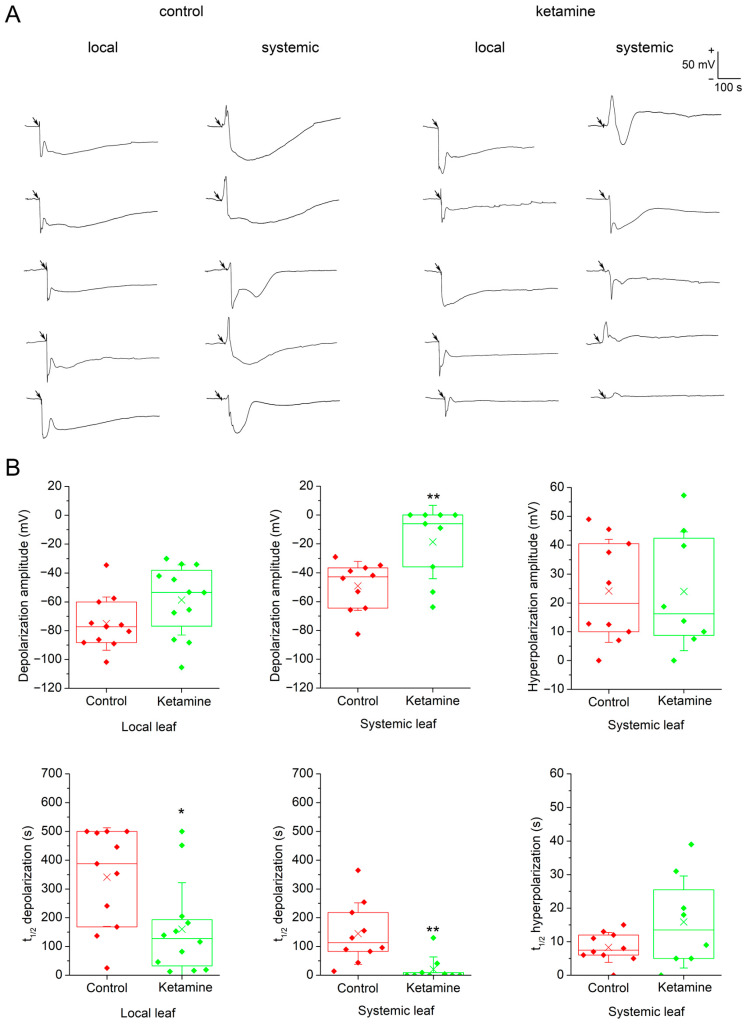
Surface potentials generated in local and systemic leaves after heat wounding in control and ketamine-treated *Arabidopsis thaliana* plants. (**A**) Example of electrical signals recorded. Time when the local leaf was heat wounded is depicted by arrows. (**B**) Amplitude and half-width duration of surface potentials in response to heat wounding in control and ketamine-treated plants in local and systemic leaves of *Arabidopsis thaliana*. Boxplots represent the 25th percentile, median, 75th percentile of the data points (diamonds). The crosses represent means, whiskers ± 1 S.D., n = 8–12. The asterisks indicate significant differences at *p* < 0.01 (**) and *p* < 0.05 (*), Welch’s-test. The terminology used (depolarisation/hyperpolarisation) is from intracellular point of view.

We used surface potential measurements for the detection of electrical signals in *A. thaliana*. One electrode was attached on a local leaf and the second one on an adjacent systemic leaf. Heat wounding was applied to the local leaf through a central vein using hot tweezers and the response was recorded. Heat wounding in the local leaf induced the hyperpolarisation of the membrane potential (a negative voltage shift recorded extracellularly, representing intracellular depolarisation), representing slow wave potential (SWP). Within several seconds after heat wounding, the electrical signal was transmitted to the systemic leaves. Before transient hyperpolarisation (representing intracellular depolarisation), some systemic leaves induced transient depolarisation (representing intracellular hyperpolarisation) of the membrane potential. The variations in electrical signal shape and intensity are shown in Figure 1A. After ketamine application, heat wounding induced a similar electrical signal in the local leaf with the same amplitude, but with slightly reduced half-time (t_1/2_). The systemic leaves received the electrical signals within a few tens of seconds with significantly reduced amplitude and t_1/2_ (Figure 1B). Sometimes, the hyperpolarisation (intracellular depolarisation) was absent and only the transient depolarisation (representing intracellular hyperpolarisation) was recorded.

### 2.2. Anaesthetic Ketamine Did Not Block Ca^2+^ Wave Propagation

**Figure 2:**
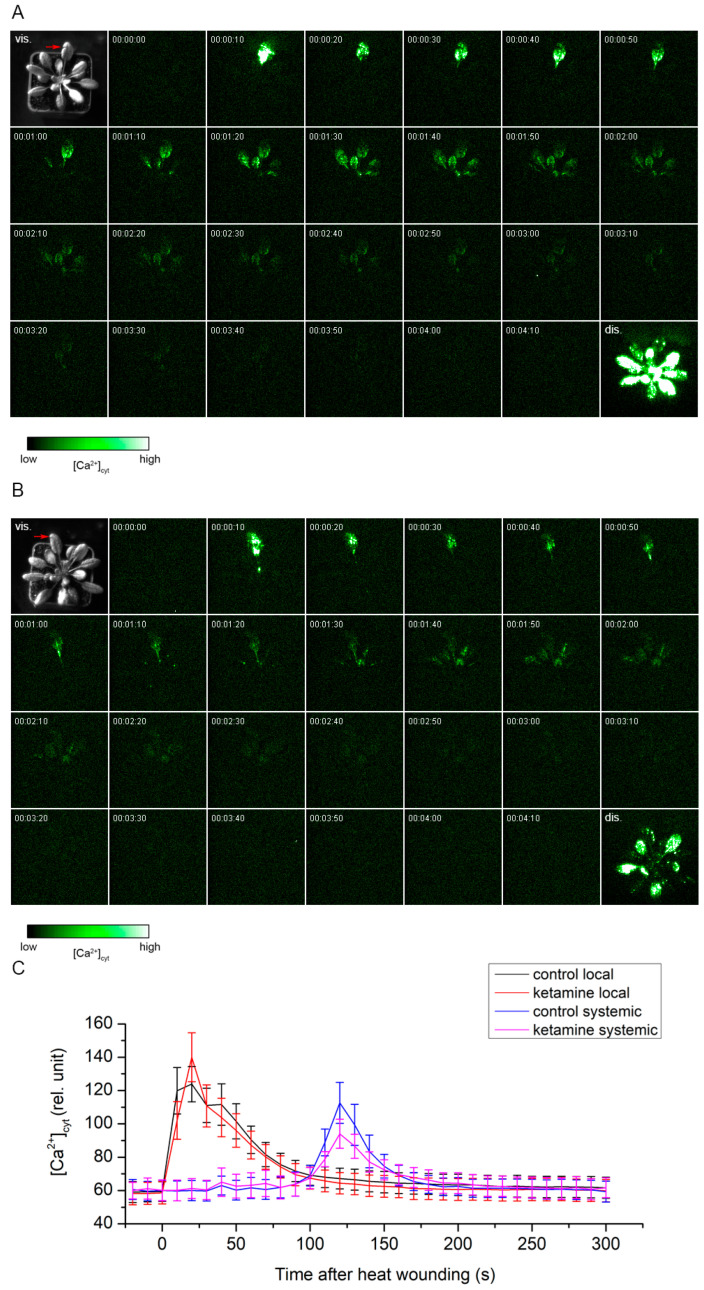
Local and systemic [Ca^2+^]_cyt_ signals in transgenic *Arabidopsis thaliana* expressing apoaequorin in response to heat wounding. (**A**) Control plants, (**B**) Ketamine-treated plants. First image in the sequence is the photograph of rosette in visible light (vis.) and the site of heat wounding across a midrib of local leaf is marked with a red arrow. Time course (00:00:00–00:04:10, h:min:s) of [Ca^2+^]_cyt_ accumulation in response to heat wounding in local and distal leaves. Heat wounding was performed in the range of 0–10 s on local leaf (frame exposure time is 10 s). Cumulative image of [Ca^2+^]_cyt_-dependent photon counts after discharge of the whole rosette (dis., frame exposure time is 1 min). Representative images from 7 to 9 independent measurements where systemic response was detected. Movies are available as Supplementary Materials, Videos S1 and S2. (**C**) Average luminescence signal intensity in local and systemic leaves (the plants where the systemic response was not detected were not involved in averaging), means ± S.E., n = 7–9. Significant differences at the same time points between control and ketamine-treated plants were not found (Welch’s *t*-test).

**Figure 3:**
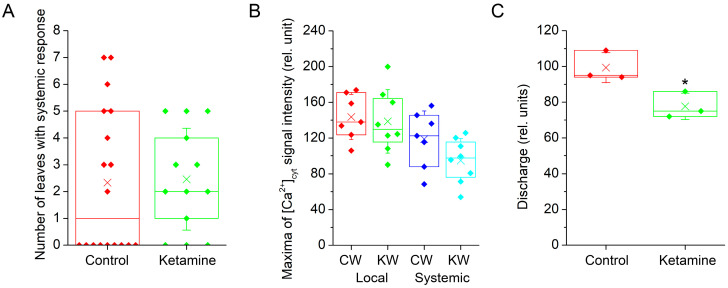
Quantification of [Ca^2+^]_cyt_ signal in transgenic *Arabidopsis thaliana* expressing apoaequorin. (**A**) Number of leaves with systemic response. Although local response was detected every time, the systemic response was not (in this case, number of systemic leaves is equal to 0), n = 13–18. (**B**) Maximum luminescence signal intensity in local and systemic leaves (the plants where the systemic response was not detected were not involved in averaging), means ± S.E., n = 7–8. (**C**) Intensity of discharge with 1 M CaCl_2_ in 10% ethanol, n = 3. Boxplots represent the 25th percentile, median, 75th percentile of the data points (diamonds). The crosses represent means, whiskers ± 1 S.D. C—control plants, K—ketamine-treated plants, W—heat-wounded plants. Significant differences between control and ketamine-treated plants were evaluated by Welch’s-test at *p* < 0.05 (*).

Because electrical and Ca^2+^ signals are tightly coupled, we further monitored [Ca^2+^]_cyt_. For this type of experiment, we used transgenic *A. thaliana* plants expressing apoaequorin from jellyfish *Aequorea victoria*. Heat wounding clearly induced a Ca^2+^ wave in the control as well as in the ketamine-treated plants (Figure 2A–C, Supplementary Materials, Videos S1 and S2). The number of systemic leaves which received electrical signals from the local wounded leaf was not significantly different between both groups of plants (Figure 3A). The maximum [Ca^2+^]_cyt_ signal intensity was comparable in local leaves but almost significantly diminished (*p* = 0.06) in the systemic leaves of ketamine-treated plants relative to the control plants (Figure 3B). But the intensity of the signal drop (20%) is comparable to the intensity of the signal drop after discharge in ketamine-treated plants (22%, Figure 3C), indicating that the Ca^2+^ wave is not significantly affected in the local or in the systemic leaves and ketamine slightly inhibited the aequorin system.

### 2.3. Ketamine Did Not Block Expression of Jasmonate Responsive Genes

**Figure 4:**
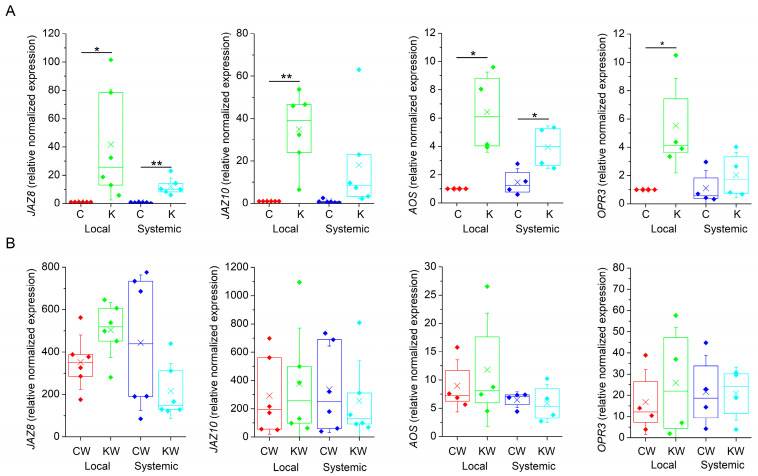
Expression of jasmonate-responsive genes in *Arabidopsis thaliana*. (**A**) Gene expression in local and systemic leaves in response to ketamine application. (**B**) Gene expression in response to heat wounding. Expression data are normalised to *ubiquitin-conjugating enzyme* (*UBC21*) n = 4–6. Boxplots represent the 25th percentile, median, 75th percentile of the data points (diamonds). The crosses represent means, whiskers ± 1 S.D. C—control plants, K—ketamine-treated plants, W—heat-wounded plants. Significant differences between control and ketamine-treated plants were evaluated by Welch’s-test at *p* < 0.01 (**) and *p* < 0.05 (*).

Further, we monitored the expression of marker genes involved in JA biosynthesis (*ALLENE OXIDE SYNTHASE*, *AOS*; *OPDA REDUCTASE 3*, *OPR3*) and JA signalling (*JASMONATE-ZIM DOMAIN* 8 and 10, *JAZ8*, *JAZ10*) in local and systemic leaves 1 h after heat wounding. Surprisingly, the application of ketamine in non-wounded plants induced the expression of all tested JA-responsive genes in comparison to control water sprayed plants (Figure 4A). As expected, wounding also increased the expression of all JA-responsive genes overall with much higher magnitude than in response to ketamine application. Ketamine-treated wounded plants exhibited comparable expression to control wounded plants indicating that ketamine had no significant effect on local and systemic JA-responsive gene expression (Figure 4B). However, on the 15^th^ day after ketamine application, the plants exhibited stunted growth and delayed development in comparison to control plants (Figure S1). Thee stunted growth and dwarf phenotype was documented in the *glr3.4*. mutant plant of rice [35]. 

## 3. Discussion

Here, we investigated the effect of the anaesthetic ketamine on electrical signal propagation in experimental model plant *A. thaliana*. These experiments were motivated by our previous research, where we found that the GVA diethyl ether completely blocked electrical and Ca^2+^ signal propagation from local to systemic leaves, induced by heat wounding or glutamate in *A. thaliana*. In response to attenuated electrical signalling, systemic leaves were not able to activate the expression of JA-responsive genes [21]. Based on these experiments, and similar experiments on Venus flytrap (*D. muscipula*) [24], the GLRs were suspected to be among the targets of diethyl ether-induced inhibition of electrical signal propagation in plants. Despite the attenuation of the Ca^2+^ wave by the GVA diethyl ether [21], the application of diethyl ether itself may induce increased cytoplasmic Ca^2+^ concentration with different spatio-temporal pattern. This affected the expression of thousands of genes indicating that the GVA diethyl ether significantly modified the calcium signature [36]. The specific molecular target of diethyl ether is not known and this anaesthetic is considered promiscuous in anaesthetic binding and may bind non-specifically to the hydrophobic pocket of many proteins [29,37] or affect channel function through its solubility in the lipid bilayer [28]. This is evidenced by its inhibition of firefly luciferase or aequorin [21,29]. In contrast, the intravenous general anaesthetic ketamine is a well-known antagonist of NMDA receptors, which are homologous to plant GLRs. However, in this case, ketamine was not able to abolish electrical and Ca^2+^ wave propagation from damaged to systemic leaves but only slightly modified their characteristics (reduced amplitude and duration, Figure 1). This modification was not sufficient to block the systemic response and systemic leaves activated the expression of JA-responsive genes (Figure 4B). Recently, it was documented that the duration of depolarisation determined the strength of jasmonate response [38]. However, in our case, the jasmonate response was not reduced and may be compensated by its activation by the ketamine itself (Figure 4A). During our experimental investigation, the cryo-electron microscope structures of human NMDA receptors in complex with ketamine were resolved. Ketamine binds in TMD in the central vestibule between the channel gate and selectivity filter. Two amino acids Leu642 on GluN2A and Asn616 on GluN1 were identified as key residues that form hydrophobic and hydrogen-bound interactions with ketamine [34]. By comparing the amino acid sequence with plant GLR3.3 and GLR3.6, it was revealed that in the position of Asn616 there is a gap and Leu642 is replaced by Asp (Figure S2). Mutated GluN2A when the Leu was substituted with Asp displayed a 226-fold reduced potency of inhibition by ketamine (IC_50_ 1.33 vs. 354.2 µM, [33]). This may indicate that if ketamine binds to the plant GLRs, it has probably reduced potency in comparison to animal neurons. On the other hand, in contrast to GVAs, ketamine is often not able to block the generation of APs in different types of animal neurons completely but only modify their characteristics (amplitude, duration, frequency, etc., [39,40,41]). All these facts may explain the weak effect of ketamine on the inhibition of electrical and Ca^2+^ signal propagation in *A. thaliana* in contrast to diethyl ether, despite the high 18 mM concentration applied. The application of a higher concentration (1%, 36 mM) exhibited leaf damage on the plants. In addition to its action on NMDA receptors, alternative targets for ketamine probably also exist and must be considered [42,43]. With its lipophilic characteristic, it may affect nonspecifically many cellular processes, like the lipophilic GVA diethyl ether [36]. Evidence of this comes from its inhibition of luminescence (Figure 3C) and the activation of JA response (Figure 4A).

Ketamine was also not effective in the inhibition of trap closure in the Venus flytrap (*D. muscipula*) [44]. We also tried to apply ketamine through the cut petiole or trap lobe, and the amplitude of AP was also not affected by 0.5% ketamine in Venus flytrap (unpublished results). In contrast, the GVA diethyl ether was able to completely abolish AP generation in the trap lobe in response to touch or wounding [22,23,24,45]. Recent studies suggested that electrical and Ca^2+^ signal propagation from the sensory cells in the sensitive trichome to the trap lobe in the Venus flytrap is probably also mediated by GLR3.6 channels and glutamate [24,46], and the electrical signal propagation from the trigger hairs to the trap lobe is analogous to the systemic signalling in the leaves of *A. thaliana* plants. The same mechanism for electrical and Ca^2+^ signal propagation may explain the similar effect of different types of anaesthetics (complete inhibition by the GVA diethyl ether, and no blockage by ketamine) on these two very different plants. The inhibition of electrical and Ca^2+^ signalling by the GVA diethyl ether in these plants may explain the blocked expression of JA-responsive genes [21,23], what was not the case in this study with ketamine. Surprisingly, ketamine, in contrast to diethyl ether, itself slightly induced the expression of JA-responsive genes (Figure 4A).

Another type of anaesthetic with a well-known molecular target is lidocaine. The mechanism of action of lidocaine as a local anaesthetic is through a blockade of voltage-gated sodium channels (VGSCs) leading to a reversible block of action potential propagation [47]. Despite the fact that plants do not have voltage-gated Na^+^ channels [48], the seismonastic plant (*Mimosa pudica*) is sensitive to the local anaesthetic lidocaine applied through the roots. Leaves of *M. pudica* fold in response to touch and other stimuli; however, this response is lost under lidocaine anaesthesia [22,49]. However, it is still not clear if lidocaine inhibited AP propagation or only the leaf-folding response, because measurement of electrical signals has not been performed. In addition to electrical signals, recent success in the transformation of *M. pudica* plants with the calcium reporter system GCaMP6f could be very helpful in this investigation [50]. Surprisingly, the Venus flytrap is not sensitive to lidocaine treatment [44]. These studies indicate that even anaesthetics with well-known defined targets probably have an off-target effect. In the case of lidocaine, such targets involve potassium and calcium channels and even NMDA receptors but usually in above clinically relevant plasma concentrations [47]. At high concentrations with their lipophilic character, they may also interact with plasma membranes (lipid theory of general anaesthesia, [26,27,28]).

The effect of GVA and local anaesthetics on plants is not only confined to the attenuation of electrical and Ca^2+^ signal propagation. They inhibit germination, chlorophyll biosynthesis, circumnutation movement of climbing plants, affect ROS homeostasis and vesicle trafficking [22]. Recently, we have shown that the GVA diethyl ether inhibited de-etiolation and the response to light stimulus in barley (*Hordeum vulgare*) seedlings. Seedlings exposed to the GVA diethyl ether had significantly decreased chlorophyll concentration and a hampered light signalling cascade, as they were not able to activate the expression of many nuclear-encoded light-responsive genes [51]. Interestingly, the application of an antagonist of animal kainate/AMPA iGluRs 6,7-dinitroquinoxaline-2,3-dione (DNQX) also impaired the light-signal transduction and phenocopy in *Arabidopsis* long-hypocotyl (*hy*) mutants [10]. From this point of view, it would be interesting to investigate the effect of ketamine on light-induced signalling pathways. 

## 4. Materials and Methods

### 4.1. Plant Material and Experimental Setup

Six-week-old *Arabidopsis thaliana* (L.) Heynh. Columbia-0 wild-type and transgenic *A. thaliana* (L.) Heynh. Columbia-0, expressing the apoaequorin gene from jellyfish *Aequorea victoria* under control of the CaMV 35S promoter, were grown on a soil substrate (Potgrond H; Klasmann-Deilmann GmbH, Geeste, Germany) in a growth chamber (AR75L; Percival-Scientific; Perry, IA, USA) set to an 8 h: 16 h photoperiod at 100 μmol m^−2^ s^−1^ PAR, 21 °C:21 °C, day/night, and 60% relative air humidity in plastic pots. Three hours before analysis, the entire plant rosettes were sprayed with 0.5% (18 mM) ketamine (racemic mixture, Lipomed AG, Arleshem, Switzerland, Catalog No. C2944, KET-663-HC) in 0.01% Tween 20 (Sigma Aldrich, St. Louis, MO, USA, Catalog No. 93773). Control plants were sprayed with distilled water with 0.01% Tween 20.

### 4.2. Extracellular Measurements of Electrical Signals

Surface potential was measured using a non-invasive non-polarizable Ag–AgCl electrodes (Scanlab systems, Prague, Czech Republic) and moistened with a drop of conductive EV gel (Hellada, Prague, Czech Republic) commonly used in electrocardiography inside a Faraday cage under standard laboratory conditions [52]. Measuring electrodes were placed at the base of the leaf blade in local and adjacent systemic leaves. The reference electrode was immersed into a basin with water, which was placed below the pot containing the measured plant. The apical part of the local leaf was wounded with hot tweezers and surface potentials were measured. The electrodes were connected to an amplifier (gain: 1–1000, noise: 2–3 mV, bandwidth (−3 dB): 10^5^ Hz, response time: 10 µs, input impedance: 10^12^ Ω) and the data were collected every 30 ms. For experimental setup and calculation of depolarisation and hyperpolarisation amplitude and half-time (t_1/2_), see our previous publication Jakšová et al. [21].

### 4.3. Aequorin Luminescence Imaging

Transgenic *A. thaliana* (L.) Heynh. Col-0 wild-type expressing the apoaequorin gene under control of the CaMV 35S promoter, was used for monitoring cytosolic free calcium [Ca^2+^]_cyt_ [53]. Aequorin was reconstituted by spraying plants with 10 μM coelenterazine (Invitrogen, Eugene, OR, USA, Catalog No. C2944) in 0.01% Tween 20 (Sigma Aldrich, St. Louis, MO, USA, Catalog No. 93773) and subsequent incubation overnight in the dark. Aequorin luminescence imaging was performed using a highly sensitive CCD camera VersArray 1300B (Princeton Instruments, Trenton, NJ, USA). To reduce the dark current, CCD camera was cooled down to −100 °C using a liquid-nitrogen cooling system. The CCD camera was equipped with a 50 mm focal distance lens with an f-number of 1.2 (Nikon, Tokyo, Japan) to enhance the light collecting efficiency. Spectral sensitivity of CCD camera was within the range of λ = 200–1000 nm with almost 90% quantum efficiency in the visible range of the spectrum. The spectral sensitivity was limited to λ = 350–1000 nm by the lenses. CCD camera parameters were as follows: scan rate, 100 kHz; gain, 2. Photons were captured in photon-counting mode with a 10 s acquisition time. Signal acquisition and processing were performed with WinView (Princeton Instruments, Trenton, NJ, USA) and ImageJ 1.49 (NIH, Bethesda, MD, USA), respectively. After each treatment, the remaining aequorin was discharged by spraying the rosette with discharge solution (1 M CaCl_2_ in 10% ethanol) while imaging aequorin luminescence with 1 min acquisition time. The CCD camera was situated in the experimental dark room (3 × 1.5 × 2.5 m) painted in black. The door in the experimental dark room was protected completely with a black curtain to restrict any external light. All experiments were repeated several times to ensure reproducibility.

### 4.4. qPCR

Control and ketamine-treated non-wounded plants, as well as heat-wounded plants were used in the experiment. Local and adjacent systemic leaves were harvested 1 h after heat wounding simultaneously with leaves from control and ketamine-treated non-wounded plants, immediately frozen in liquid nitrogen and stored at −80 °C before gene expression analyses. This time point was chosen based on our previous analyses [21]. RNA was extracted using Spectrum Plant Total RNA kit (Sigma–Aldrich, St. Louis, MO, USA, Catalog No. STRN50) and DNase I treated and purified by RNA Clean & Concentrator Kit (Zymoresearch, Irvine, CA, USA, Catalog No. R1013) according to manufacturer’s instructions. The concentration and sample purity were measured by NanoReady (LifeReal, Hangzhou, China). The synthesis of first strand of cDNA was performed by iScript™ cDNA Synthesis Kit (BIO-RAD, Hercules, CA, USA, Catalog No. 1708890) using manufacturer’s protocol. cDNA samples were 10-times diluted prior to qPCR run. For real-time PCR, specific gene sequences were amplified by Maxima SYBR Green/ROX qPCR Master Mix (Thermo Fisher Scientific, Haltman, MA, USA, Catalog No. K0221). Primers for amplification of housekeeping gene sequence (*UBIQUITIN-CONJUGATING ENZYME—UBC21*) and sequence for gene of interest (*JASMONATE-ZIM DOMAIN 8*, *JAZ8*; *JASMONATE-ZIM DOMAIN 10*, *JAZ10*; *OPDA REDUCTASE 3*, *OPR3*; *ALLENE OXIDE SYNTHASE*, *AOS*) were used according to [4,54] (Table S1). Real-time PCR reactions were performed in 96-well plates in triplicate on a QuantStudio 5 Real-Time PCR System (Applied Biosystems, Haltman, MA, USA) device and the relative changes in gene expression were estimated according to Pfaffl [55]. Melt curve analysis of qPCR products was included at the end of each qPCR run to verify product specificity. All samples for PCR experiments were analysed in four to six biological and three technical replicates.

### 4.5. Statistical Analysis

All data are from biological replicates. Data are shown as means ± S.D. or box plots. Before statistical analyses, the data were tested for homogeneity of variance (Brown–Forsythe test). If the homogeneity was fulfilled, Student’s *t*-test was used (Origin 8.5.1, Northampton, MA, USA). If homogeneity was not present, Welch’s test was used (Microsoft Excel 2016).

## 5. Conclusions

This study showed that the general anaesthetic ketamine did not inhibit the systemic response in *A. thaliana* in comparison to the effect of the GVA diethyl ether [21]. Diethyl ether is promiscuous in anaesthetic binding and may affect membrane fluidity, different proteins, cytoskeleton, etc., [37]. On the other hand, ketamine, considered as an anaesthetic with a well-known binding site for NMDA receptors, is probably not able to sufficiently inhibit the plant’s GLR due to the absence of the conserved amino acid residues necessary for ketamine binding. From our study, it is not clear if the modification of electrical signals and stunted growth in response to ketamine application is specific to GLR action or some pleiotropic effect caused by its lipophilic characteristic. Indeed, alternative targets probably exist and must be considered besides its action on NMDA receptors [43]. Therefore, we must be very careful in interpreting GLR function based on comparison with its animal counterparts, as has been suggested by Wudick et al. [12]. The use of well-known agonists and antagonists of animal iGluR [10] must be performed with caution in plant models.

## Data Availability

Upon request, the data will be provided by the corresponding author.

## Supplementary Materials

The following supporting information can be downloaded at: https://www.mdpi.com/article/10.3390/plants13060894/s1, Figure S1: The plants of *Arabidopsis thaliana* 24 h (A) and 15 days (B) after experiments with ketamine application; Figure S2: Amino acid alignment of human glutamate receptor NMDA 2A isoform 1 (NP_000824.1) and *Arabidopsis thaliana* GLR3.3 (NP_001322169.1) and GLR 3.6 (NP_001326369.1); Table S1: Primers sequences used in qPCR; Video S1: The [Ca^2+^]_cyt_ signals in transgenic *Arabidopsis thaliana* expressing apoaequorin in response to heat wounding in control plants; Video S2: The [Ca^2+^]_cyt_ signals in transgenic *Arabidopsis thaliana* expressing apoaequorin in response to heat wounding in ketamine-treated plants.

## References

[1] Koo A.J.K., Howe G.A. (2009). The wound hormone jasmonate. Phytochemistry.

[2] Wasternack C., Hause B. (2013). Jasmonates: Biosynthesis, perception, signal transduction and action in plant stress response, growth and development. An update to the 2007 review in Annals of Botany. Ann. Bot..

[3] Schilmiller A.L., Howe G.A. (2005). Systemic signaling in the wound response. Curr. Opin. Plant Biol..

[4] Mousavi S.A.R., Chauvin A., Pascaud F., Kellenberger S., Farmer E.E. (2013). GLUTAMATE RECEPTOR-LIKE genes mediate leaf-to-leaf wound signalling. Nature.

[5] Gilroy S., Suzuki N., Miller G., Choi W.-G., Toyota M., Devireddy A.R., Mittler R. (2014). A tidal wave of signals: Calcium and ROS at the forefront of rapid systemic signaling. Trends Plant Sci..

[6] Gilroy S., Białasek M., Suzuki N., Górecka M., Devireddy A.R., Karpinski S., Mittler R. (2016). ROS, calcium, and electric signals: Key mediators of rapid systemic signaling in plants. Plant Physiol..

[7] Toyota M., Spencer D., Sawai-Toyota S., Jiaqi W., Zhang T. (2018). Glutamate triggers long-distance, calcium-based plant defense signaling. Science.

[8] Suda H., Toyota M. (2022). Integration of long-range signals in plants: A model for wound-induced Ca^2+^, electrical, ROS, and glutamate waves. Curr. Opin. Plant Biol..

[9] Wildon D.C., Thain J.F., Minchin P.E.H., Gubb I.R., Reilly A.J., Skipper Y.D., Doherty H.M., O’Donnell P.J., Bowles D.J. (1992). Electrical signalling and systemic proteinase inhibitor induction in the wounded plant. Nature.

[10] Lam H.M., Chiu J., Hsieh M.H., Meisel L., Oliveira I.C., Shin M., Coruzzi G. (1998). Glutamate-receptor genes in plants. Nature.

[11] Price M.B., Jelesko J., Okumoto S. (2012). Glutamate receptor homologs in plants: Functions and evolutionary origins. Front. Plant Sci..

[12] Wudick M.M., Michard E., Nunes C.O., Feijó J.A. (2018). Comparing plant and animal glutamate receptors: Common traits but different fates?. J. Exp. Bot..

[13] Nguyen C.T., Kurenda A., Stolz S., Chételat A., Farmer E.E. (2018). Identification of cell populations necessary for leaf-to leaf electrical signaling in a wounded plant. Proc. Natl. Acad. Sci. USA.

[14] Alfieri A., Doccula F.G., Pederzoli R., Grenzi M., Bonza M.C., Luoni L., Candeo A., Armada N.R., Barbiroli A., Valentini G. (2020). The structural bases for agonist diversity in an *Arabidopsis thaliana* glutamate receptor-like channel. Proc. Natl. Acad. Sci. USA.

[15] Shao Q., Gao Q., Lhamo D., Zhang H., Luan S. (2020). Two glutamate- and pH-regulated Ca^2+^ channels are required for systemic wound signaling in *Arabidopsis*. Sci. Signal..

[16] Grenzi M., Bonza M.C., Alfieri A., Costa A. (2021). Structural insights into long-distance signal transduction pathways mediated by plant glutamate receptor-like channels. New Phytol..

[17] Grenzi M., Bonza M.C., Costa A. (2022). Signaling by plant glutamate receptor-like channels: What else!. Curr. Opin. Plant Biol..

[18] Moe-Lange J., Gappel N.M., Machado M., Wudick M.M., Sies C.S., Schott-Verdugo S.N., Bonus M., Mishra S., Hartwig T., Bezrutczyk M. (2021). Interdependence of a mechanosensitive anion channel and glutamate receptors in distal wound signaling. Sci. Adv..

[19] Bellandi A., Papp D., Breakspear A., Joyce J., Johnston M.G., de Keijzer J., Raven E.C., Ohtsu M., Vincent T.R., Miller A.J. (2022). Diffusion and bulk flow of amino acids mediate calcium waves in plants. Sci. Adv..

[20] Grenzi M., Buratti S., Parmagnani A.S., Aziz I.A., Bernacka-Wojcik I., Resentini F., Šimura J., Doccula F.G., Alfieri A., Luoni L. (2023). Long-distance turgor pressure changes induce local activation of plant glutamate receptor-like channels. Curr. Biol..

[21] Jakšová J., Rác M., Bokor B., Petřík I., Novák O., Reichelt M., Mithöfer A., Pavlovič A. (2021). Anaesthetic diethyl ether impairs long-distance electrical and jasmonate signaling in *Arabidopsis thaliana*. Plant Physiol. Biochem..

[22] Yokawa K., Kagenishi T., Pavlovič A., Gall S., Weiland M., Mancuso S., Baluška F. (2018). Anaesthetics stop diverse plant organ movements, affect endocytic vesicle recycling and ROS homeostasis, and block action potentials in Venus flytraps. Ann. Bot..

[23] Pavlovič A., Libiaková M., Bokor B., Jakšová J., Petřík I., Novák O., Baluška F. (2020). Anaesthesia with diethyl ether impairs jasmonate signalling in the carnivorous plant Venus flytrap (*Dionaea muscipula*). Ann. Bot..

[24] Scherzer S., Huang S., Iosip A., Kreuzer I., Yokawa K., Al-Rasheid K.A.S., Heckmann M., Hedrich R. (2022). Ether anesthetics prevents touch-induced trigger hair calcium-electrical signals excite the Venus flytrap. Sci. Rep..

[25] MacIver B.M., Tanelian D.L. (1990). Volatile anesthetics excite mammalian nociceptor afferents recorded in vitro. Anesthesiology.

[26] Meyer H. (1899). Zur theorie der Alkoholnarkose. Arch. Exp. Pathol. Phar..

[27] Overton C.E. (1901). Studien über die Narkose Zugleich ein Beitrag zur Allgemeinen Pharmakologie.

[28] Pavel M.A., Petersen N., Wang H., Lerner R.A., Hansen S.B. (2020). Studies on the mechanism of general anesthesia. Proc. Natl. Acad. Sci. USA.

[29] Franks N.P., Lieb W.R. (1984). Do general anesthetics act by competitive binding to specific receptors?. Nature.

[30] Franks N.P. (2006). Molecular targets underlying general anesthesia. Br. J. Pharmacol..

[31] Crowder C.M. (2008). Does natural selection explain the universal response of metazoans to volatile anesthetics?. Anesth. Analg..

[32] Eckenhoff R.G. (2008). Why can all of biology be anesthetized?. Anesth. Analg..

[33] Franks N.P. (2008). General anaesthesia: From molecular targets to neuronal pathways of sleep and arousal. Nat. Rev. Neurosci..

[34] Zhang Y., Ye F., Zhang T., Lv S., Zhou L., Du D., Lin H., Guo F., Luo C., Zhu S. (2021). Structural basis of ketamine action on human NMDA receptors. Nature.

[35] Yu B., Wu Q., Li X., Zeng R., Min Q., Huang J. (2022). GLUTAMATE RECEPTOR-like gene OsGLR3.4 is required for plant growth and systemic wound signaling in rice (*Oryza sativa*). New Phytol..

[36] Pavlovič A., Jakšová J., Kučerová Z., Špundová M., Rác M., Roudnický P., Mithöfer A. (2022). Diethyl ether anesthesia induces transient cytosolic [Ca^2+^] increase, heat shock proteins, and heat stress tolerance of photosystem II in *Arabidopsis*. Front. Plant Sci..

[37] Kelz M.B., Mashour G.A. (2019). The biology of general anesthesia from paramecium to primate. Curr. Biol..

[38] Kumari A., Chételat A., Nguyen C.T., Farmer E.E. (2019). Arabidopsis H^+^-ATPase AHA1 controls slow wave potential duration and wound-response jasmonate pathway activation. Proc. Natl. Acad. Sci. USA.

[39] Marwaha J. (1980). Some mechanisms underlying actions of ketamine on electromechanical coupling in skeletal muscle. J. Neurosci. Res..

[40] MacDonalds J.F., Bartlett M.C., Mody I., Pahapill P., Reynolds J.N., Salter M.W., Schneiderman J.H., Pennefather P.S. (1991). Actions of ketamine, phencyclidine and MK-801 on NMDA receptor currents in cultured mouse hippocampal neurones. J. Physiol..

[41] Hatakeyama N., Yamazaki M., Shibuya N., Yamamura S., Momose Y. (2001). Effects of ketamine on voltage-dependent calcium currents and membrane potentials in single bullfrog atrial cells. J. Anesth..

[42] Yin J., Fu B., Wang Y., Yu T. (2019). Effects of ketamine on voltage-gated sodium channels in the barrel cortex and the ventral posteromedial nucleus slices of rats. Neuroreport.

[43] Zorumski C.F., Izumi Y., Mennerick S. (2016). Ketamine: NMDA receptors and beyond. J. Neurosci..

[44] De Luccia T.P. (2012). *Mimosa pudica*, *Dionaea muscipula* and anesthetics. Plant Signal. Behav..

[45] Böhm J., Scherzer S. (2021). Signaling and transport processes related to the carnivorous lifestyle of plants living on nutrient-poor soil. Plant Physiol..

[46] Iosip A.L., Böhm J., Scherzer S., Al-Rasheid K.A.S., Dreyer I., Schultz J., Becker D., Kreuzer I., Hedrich R. (2020). The Venus flytrap trigger hair-specific potassium channel KDM1 can reestablish the K^+^ gradient required for hapto-electric signaling. PLoS Biol..

[47] Hermanns H., Hollmann M.W., Stevens M.F., Lirk P., Brandenburger T., Piegeler T., Werdehausen R. (2019). Molecular mechanisms of action of systemic lidocaine in acute and chronic pain: A narrative review. Brit. J. Anaesth..

[48] Hedrich R. (2012). Ion channels in plants. Physiol Rev..

[49] Milne A., Beamish T. (1999). Inhalational and local anesthetics reduce tactile and thermal responses in *Mimosa pudica*. Can. J. Anaesth..

[50] Hagihara T., Mano H., Miura T., Hasebe M., Toyota M. (2022). Calcium-mediated rapid movements defend against herbivorous insects in *Mimosa pudica*. Nat. Commun..

[51] Pavlovič A., Kopečná M., Hloušková L., Koller J., Hřivňacký M., Ilík P., Bartoš J. (2024). Diethyl ether anaesthesia inhibits de-etiolation of barley seedlings by locking them in intermediate skoto-photomorphogenetic state. Physiol. Plantarum.

[52] Ilík P., Hlaváčková V., Krchňák P., Nauš J. (2010). A low-noise multi-channel device for the monitoring of systemic electrical signal propagation in plants. Biol. Plantarum.

[53] Kiep V., Vadassery J., Lattke J., Maaβ J.-P., Boland W., Peiter E., Mithöfer A. (2015). Systemic cytosolic Ca^2+^ elevation is activated upon wounding and herbivory in *Arabidopsis*. New Phytol..

[54] Chini A., Monte I., Zamarreño A.M., Hamberg M., Lassuer S., Reymond P., Weiss S., Stintzi A., Schaller A., Porzel A. (2018). An OPR3-independent pathway uses 4,5-didehydrojasmonate for jasmonate synthesis. Nat. Chem. Biol..

[55] Pfaffl M.W. (2001). A new mathematical model for relative quantification in real-time RT–PCR. Nucleic Acids Res..

