# Supplementary material for: Effect of the General Anaesthetic Ketamine on Electrical and Ca2+ Signal Propagation in Arabidopsis thaliana
Source: Plants (Basel). 2024 Mar 20;13(6):894. doi: 10.3390/plants13060894 (PMC10975207; doi:10.3390/plants13060894)
Supplement: Supplementary file 1 [file plants-13-00894-s001.zip › Supplementary Materials.pdf]

SUPPLEMENTARY MATERIAL

Effect of the general anaesthetic ketamine on electrical and Ca^{2+} signal propagation in the *Arabidopsis thaliana*.

Andrej Pavlovič[1*], Lucie Ševčíková[1], Martin Hřivňacký[1], Marek Rác[1]

[1]*Department of Biophysics, Faculty of Science, Palacký University, Šlechtitelů 27, CZ-783 71, Olomouc, Czech Republic.*

Figure S1 The plants of *Arabidopsis thaliana* 24 h (A) and 15 days (B) after experiments with ketamin application.

Figure S2 Aminoacid alignment of human glutamate receptor NMDA 2A isoform 1 (NP_000824.1) and *Arabidopsis thaliana* GLR3.3 (NP_001322169.1) and GLR 3.6 (NP_001326369.1).

Movie S1 The $[Ca^{2+}]_{cyt}$ signals in transgenic *Arabidopsis thaliana* expressing APOAEQUORIN in response to heat wounding in control plants

Movie S2 The $[Ca^{2+}]_{cyt}$ signals in transgenic *Arabidopsis thaliana* expressing APOAEQUORIN in response to heat wounding in ketamine-treated plants.

Table S1 Primers sequences used in qPCR.

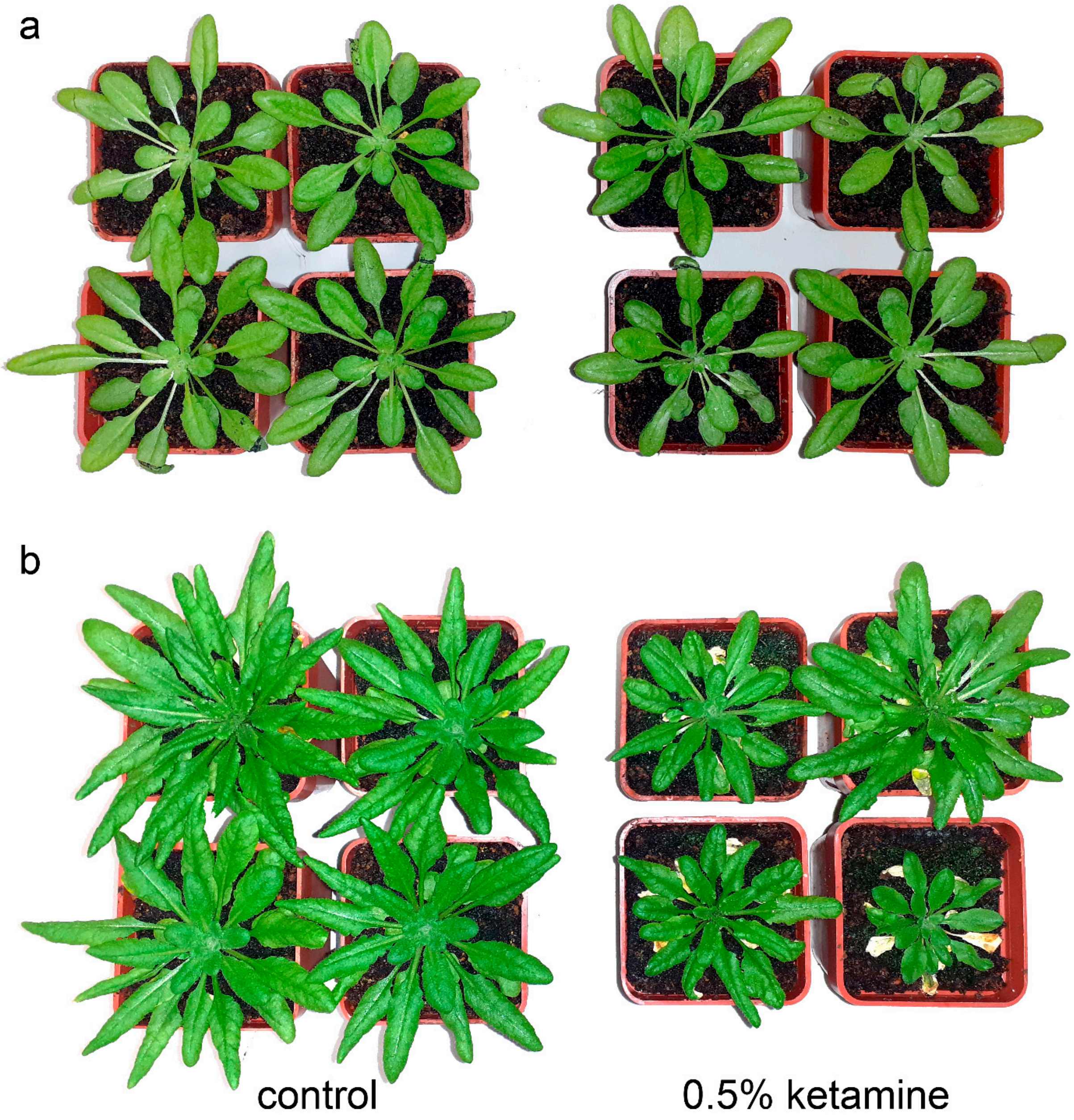

Figure S1 The plants of *Arabidopsis thaliana* 24 h (A) and 15 days (B) after experiments with ketamin application.

glutamate receptor 3.3 [Arabidopsis thaliana]

Sequence ID: NP_001322169.1 Length: 933 Number of Matches: 2

See 7 more title(s) See all Identical Proteins(IPG)

Range 1: 491 to 847 GenPept Graphics ▼ Next Match ▲ Previou

Score	Expect	Method	Identities	Positives	Gaps
84.3 bits(207)	2e-20	Compositional matrix adjust.	84/385(22%)	165/385(42%)	34/385(8%)

```
Query  457  KGFCIDILKKLSRTVKFTYDLYLVTNGKHGKKVNNVWNGMIGEVVYQRAVMAVGSLTINE  516
            KGFCID+       + +   +  +  G +GK+ N  +  M+  +        VG + I
Sbjct  491  KGFCIDVFTAAVNLLPYAVPVKFIPYG-NGKE-NPSYTHMVEMITTGNFDGVVGDVAIVT  548

Query  517  ERSEVVDFSVPFVETGISVMVSRSNGTVSPSAFLEPFSASVWVMMFVMLLIVSAIAVFVF  576
             R+++VDF+ P+  +G+ V+           AFL PF+  +W +     L V  I V++
Sbjct  549  NRTKIVDFTQPYAASGLVVVAPFKKLNSGAWAFLRPFNRLMWAVTGCCFLFV-GIVVWIL  607

Query  577  EYFSPVGYNRNLAKGKAPHGPSFTIGKAIWLLWGLVFNNSVPVQNPKGTTSKIMVSVWAF  636
            E+       R   + + P  P       +W  +  +F      +N   T  ++++ +W F
Sbjct  608  EH-------RTNDEFRGP--PKRQCVTILWFSFSTMF--FAHRENTVSTLGRLVLIIWLF  656

Query  637  FAVIFLASYTANLAAFMIQEEFVDQVTGLSDKKFQRPHDYSPPFRFGTVPNGSTERNIRN  696
              +I  +SYTA+L + +  ++    + G+      R  D    ++ G+      E  +RN
Sbjct  657  VVLIINSSYTASLTSILTVQQLSSPIKGIES---LRERDDPIGYQVGSF----AESYLRN  709

Query  697  NYPYMHQYMTKFNQKGVEDALV-SLKTGKLDAFIYDAAVLNYKAGRD----EGCKLVTIG  751
                ++   ++    G  +A   +LK G     +  AA+++ +   +      C    +G
Sbjct  710  E---LNISESRLVPLGTPEAYAKALKDGPSKGGV--AAIVDERPYVELFLSSNCAYRIVG  764

Query  752  SGYIFATTGYGIALQKGSPWKRQIDLALLQFVGDGEMEELETLWLT-GICHNEKNEVMSS  810
                F  +G+G A  + SP    +  A+L    +G+++ +   WL    C  E  E+ S
Sbjct  765  QE--FTKSGWGFAFPRDSPLAIDLSTAILELAENGDLQRIHDKWLMKNACTLENAELESD  822

Query  811  QLDIDNMAGVFYMLAAAMALSLITF  835
            +L + +  G+F +   A  L+L  +
Sbjct  823  RLHLKSFWGLFLICGVACLLALFLY  847
```

glutamate receptor 3.6 [Arabidopsis thaliana]

Sequence ID: NP_001326369.1 Length: 903 Number of Matches: 1

See 5 more title(s) See all Identical Proteins(IPG)

Range 1: 458 to 837 GenPept Graphics ▼ Next Match ▲ Previous Match

Score	Expect	Method	Identities	Positives	Gaps
94.4 bits(233)	2e-23	Compositional matrix adjust.	100/420(24%)	186/420(44%)	54/420(12%)

```
Query  430  VRNTVPCR-KFVKINNSTNEGMNVKKCCKGFCIDILKKLSRTVKFTYDLYLVTNGK-HGK  487
            +R  VP R +F ++ +  + GM       GFC+D+       + +     LV  G  H
Sbjct  458  LRIGVPNRYRFEEVVSVKSNGM-----ITGFCVDVFIAAINLLPYAVPFELVAFGNGHDN  512

Query  488  KVNNVWNGMIGEVVYQRAVMAVGSLTINEERSEVVDFSVPFVETGISVMVSRSNGTVSPS  547
              N+    +I   VY      VG +TI  ER+++ DF+ P+VE+G+ V+        S
Sbjct  513  PSNSELVRLITTGVYD---AGVGDITIITERTKMADFTQPYVESGLVVVAPVRKLGSSAM  569

Query  548  AFLEPFSASVWVMMFVMLLIVSAIAVFVFEYFSPVGYNRNLAKGKAPHGPSFTIGKAIWL  607
            AFL PF+  +W++     LIV A+ ++  E+       ++  + + P  P   +    W
Sbjct  570  AFLRPFTPQMWLIAAASFLIVGAV-IWCLEH-------KHNDEFRGP--PRRQVITTFWF  619

Query  608  LWGLVFNNSVPVQNPKGTTS---KIMVSVWAFFAVIFLASYTANLAAFMIQEEFVDQVTG  664
             +  +F       + + TTS   +I++ +W F  +I  +SYTA+L + +   +    + G
Sbjct  620  SFSTLF-----FSHRETTTSNLGRIVLIIWLFVVLIINSSYTASLTSILTVHQLSSPIKG  674

Query  665  LSDKKFQRPHDYSPPFRFGTVPNGSTERNIRNNYPYMHQYMTKFNQ----KGVEDALVSL  720
            +  +  Q  HD   P  +   P GS  R+       +H+     ++    +  E+   +L
Sbjct  675  I--ETLQTNHD---PIGY---PQGSFVRDY-----LIHELNIHVSRLVPLRSPEEYDKAL  721

Query  721  KTGKLDAFIYDAAVLNYKAGRD----EGCKLVTIGSGYIFATTGYGIALQKGSPWKRQID  776
            + G     +  AAV++ +A  +      C+   +G    F   G+G A  + SP    +
Sbjct  722  RDGPGKGGV--AAVVDERAYIELFLSNRCEFGIVGQE--FTKNGWGFAFPRNSPLAVDVS  777

Query  777  LALLQFVGDGEMEELETLWLT-GICHNEKNEVMSSQLDIDNMAGVFYMLAAAMALSLITF  835
             A+LQ   +G+M+ +   WL    C  +  E+   +L++ +  G+F +   A  L+L  +
Sbjct  778  AAILQLSENGDMQRIRDKWLLRKACSLQGAEIEVDRLELKSFWGLFVVCGVACVLALAVY  837
```

Figure S2 Aminoacid alignment of human glutamate receptor NMDA 2A (GluN2A) isoform 1 (NP_000824.1) and *Arabidopsis* thaliana GLR3.3 (NP_001322169.1) and GLR 3.6 (NP_001326369.1). The critical residues involved in ketamine binding (L642, N614) are highlighted in red rectangles (NCBI Blast).

Table S1 Primer sequences used in qPCR

Gene name	Primer sequences
JAZ8	forward 5′-TGTGTTTTTCTTCAGATGTTACCC-3′<br>reverse 5′-TCTCTGCTTGCGATCGATATT-3′
JAZ10	forward 5′-ATCCCGATTTCTCCGGTCCA-3′<br>reverse 5′-ACTTTCTCCTTGCGATGGGAAGA-3′
OPR3	forward 5′-GCATGGAAGCAAGTTGTGGAAGCA-3′<br>reverse 5'-CATGCGCCCCGTGGATCTCAAT-3′
AOS	forward 5'-GCGACGAGAGATCCGAAGA-3'<br>reverse 5'-CTCGCCACCAAAACAACAAA -3'
UBC21	forward 5′-CAGTCTGTGTGTAGAGCTATCATAGCAT-3′<br>reverse 5′-AGAAGATTCCCTGAGTCGCAGTT-3′

Movie S1 The $[Ca^{2+}]_{cyt}$ signals in transgenic *Arabidopsis thaliana* expressing apoaequorine in response to heat wounding in control plants. (a) photograph of plant rosette, (b) $[Ca^{2+}]_{cyt}$ signal, (c) discharge. The site of heat wounding across a midrib of local leaf is marked with red arrow. The leaf was heat wounded between 00:00-00:10 s.

Movie S2 The $[Ca^{2+}]_{cyt}$ signals in transgenic *Arabidopsis thaliana* expressing apoaequorine in response to heat wounding in ketamine-treated plants. (a) photograph of plant rosette, (b) $[Ca^{2+}]_{cyt}$ signal, (c) discharge. The site of heat wounding across a midrib of local leaf is marked with red arrow. The leaf was heat wounded between 00:00-00:10 s.
